# Acute and Subacute Oral Toxicity Study of a Herbal Formulation Containing *Asparagus racemosus*, *Tinospora cordifolia*, and *Trigonella foenum-graceum* in Mice

**DOI:** 10.1155/jt/8221552

**Published:** 2025-02-12

**Authors:** Saurabh Maru, Sateesh Belemkar

**Affiliations:** ^1^Department of Pharmacology, School of Pharmacy and Technology Management, SVKM's NMIMS, Shirpur 425405, Maharashtra, India; ^2^Department of Pharmacology, Shobhaben Pratapbhai Patel School of Pharmacy & Technology Management, SVKM's NMIMS, Mumbai 400056, India

**Keywords:** acute toxicity, galactagogue, no-observed-adverse-effect level (NOAEL), phytomedicine, repeated-dose toxicity, safety profile

## Abstract

**Background:** The synergistic activity of compounds in herbal drugs has been well established by multiple scientific studies. The compounds present in plants may have increased toxicity and increased efficacy. Owing to the notion that traditional medicines do not have any adverse effects, these are used heftily.

**Aim:** The present study was designed to assess the toxicity of an herbal drug consisting of *Asparagus racemosus* roots, *Tinospora cordifolia* stems, and *Trigonella foenum-graecum* seeds extract blend (ATTEB), which is widely employed as an antimicrobial, anti-inflammatory, immunomodulator, adaptogen, female tonic, galactagogue, etc.

**Methodology:** The current study evaluated its safety by acute (OECD 423) and subacute (OECD 407) repeated-dose toxicity studies. A phytochemical investigation was carried out and revealed the presence of principal bioactive constituents. A genotoxicity study was performed by micronucleus assay. Gross necroscopy of the animals was performed, and behavioral, hematological, biochemical, and histopathological studies were performed.

**Results:** In the acute toxicity study, there was no mortality and no significant changes in behavior, organ structure, or organ weight, as observed by gross necroscopy of the animals, at a single dose of 2000 mg/kg BW. In a 28-day repeated-dose toxicity study, up to a daily dose of 1000 mg/kg BW, there was no evidence of toxicity. No significant genotoxicity was observed in the mice.

**Conclusion:** The LD_50_ found to be greater than 2000 mg/kg BW with NOAEL at 1000 mg/kg BW in mice. It was found to be free from any genotoxicity. The herbal drug was found to be safe to level of category 4 and can be used further for clinical studies.

## 1. Introduction

According to estimates from the World Health Organization (WHO), primary healthcare is still mostly provided by traditional herbal remedies for 80% of the world's population [1]. These medicines have developed over generations on the basis of practical and evidence-based knowledge of their efficacy and toxicity. Owing to their high efficacy, ready availability, and low cost, these medicines are widely employed in developing and underdeveloped countries. These drugs have been shown to have few adverse and toxic effects. Thus, because these drugs are safe at any dose, a hefty consumption tendency of these drugs has been noted in the population. The WHO reported that the inappropriate use of traditional herbal medicines may cause adverse events. Thus, establishing a safety profile of these drugs alone or in combination is important to assure their safe use in the population. Systematically designed safety studies of traditional herbal medicines may increase the confidence of clinicians in their use of these medicines for patients.

In traditional practices, the majority of preparations are blends of plant extracts rather than single plant extracts. Many phytomedicinal preparations are polyherbal blends and contain several bioactive ingredients that may be difficult to characterize because they are derived from a wide variety of plant species and families. Compared with their individual components, these polyherbal blends are generally more effective in terms of biological action. In addition, along with its enhanced activity, its toxicity can be increased owing to the presence of more active components with similar effects on a particular organ [2]. Therefore, a precise scientific definition of the raw components utilized in such preparations may serve as the basis for defining quality and safety criteria for herbal medications.

*Asparagus racemosus* (ARE) belongs to the Liliaceae family and contains steroidal saponin glycosides shatavarins (I–IV) [3]; isoflavones; asparagamine; racemosol; polysaccharides; mucilage; vitamins A, B1, B2, C, and E; minerals, mainly Mg, P, Ca, and Fe; folic acid; essential oils; asparagine; arginine; tyrosine; flavonoids (kaempferol, quercetin, and rutin); resin and tannins; and oligospirostanoside (immunoside) [3–5]. It is used as a potent antioxidant, antibacterial, antimicrobial, immune stimulant, adaptogenic, antiulcerogenic, analgesic, antihepatotoxic, antidyspepsia, galactagogue, antitussive, antitumor, antiinflammation, antinuropathic, cardioprotective, and antibacterial agent and as a treatment for cough, bronchitis, and neurodegenerative disorder [3].

**Figure 1:**
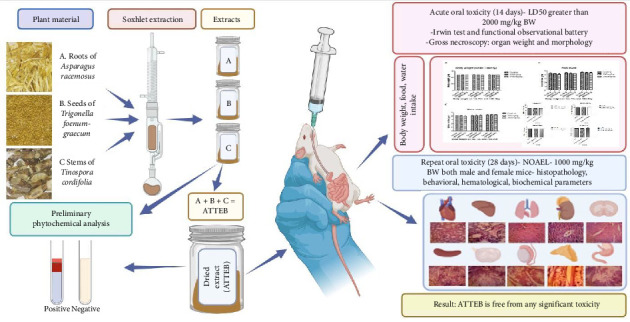
Details of the toxicity studies performed. Extract blend (ATTEB) was prepared from extracts of *Asparagus racemosus* roots, *Tinospora cordifolia* stems, and *Trigonella foenum-graecum* seeds. The extracts were tested for phytochemicals, and acute and subacute toxicity studies were performed.

In toxicity studies of ARE, no-observed-adverse-effect level (NOAEL) was reported to be greater than 2000 mg/kg/day. The lethal dose 50 (LD_50_) has been reported to be more than 2000 mg/kg/day on oral treatment, and for i.p. dosing, it is reported to be 505 mg/kg/day aqueous extract [6]. Multiple studies have reported no mortality in acute toxicity studies [7] (Figure 1).

*Tinospora cordifolia* (TCE), which belongs to the Menispermaceae family, strengthens the body's defenses against illness. The Ayurvedic Pharmacopoeia of India recognizes the medicinal use of the entire plant, but specifically the stem, because it contains more alkaloids than the leaves do. Alkaloids, namely, berberine, palmetine, and tinosporine, are present in sufficient amounts in the stem. The glycosides found are tinocordifolioside, cordifoliosides A and B, the diterpenoid lactone furanolactone, and clerodane derivatives (amritosides A, B, C, and D) [8–10]. It has been used for its general tonic, antiinflammatory, antiarthritic, antiallergic, antimalarial, antidiabetic, and aphrodisiac properties [11]. The stem is the official medicine listed by the Ayurvedic Pharmacopoeia of India. The stem of *T. cordifolia* is one of the constituents of several Ayurvedic preparations used for general debility, dyspepsia, fever, and urinary diseases. Owing to its immunomodulatory properties, *T. cordifolia* has been reported to be used successfully to treat various infections, including infections of the mammary gland [12, 13]. The LD_50_ of the ethanolic extract was reported to be 6.83 g/kg of body weight, and the extract was administered i.p. [14]. The NOAEL of aqueous extract was found to be 1000 mg/kg BW per day [15].

*Trigonella foenum-graecum* (TFGE) plants contain high levels of soluble fiber, mucilage, and galactomannan, which inhibit the digestion and absorption of starch and bile salts. Seeds of this plant contain 0.2%–0.9% diosgenin, 4.8% saponins, 35% alkaloids, and 100 mg of flavonoids per gram of fenugreek seeds [16, 17]. In addition, the steroidal sapogenin and diosgenin found in fenugreek have been shown to increase milk production in nursing mothers [18, 19]. The LD_50_ was reported to be greater than 5 g/kg body weight [20]. Body weight gain is reduced, water intake is increased, blood sugar is decreased, and ALT levels are increased [21].

The ARE roots, TCE stems, and TFGE seeds extract blend (ATTEB) are widely used by rural people on the Indian subcontinent as traditional medicines because of their multiple activities, such as antimicrobial, anti-inflammatory, immunomodulator, adaptogen, female tonic, and galactagogue activities [22–25]. A blend of these three extracts has been reported to exert synergistic effects in multiple studies, but their safety has not been established.

Thus, in the present study, a blend of hydroalcoholic extracts of ATTEB was studied for safety and assessment of possible toxicity.

## 2. Materials and Methods

### 2.1. Chemicals

All the solvents and reagents were of analytical grade and purchased from Merck, India, and Sigma-Aldrich, USA.

### 2.2. Plant Material and Extraction

The dried roots of ARE, stems of TCE, and seeds of TFGE L. were obtained from D. G. Ayurvedic Sangrah, Andheri, Mumbai. Its authenticity was certified by the Department of Botany, Govt. Holkar Science College, Indore, M.P., India, and voucher specimens were deposited in the department for future reference.

For extraction, the collected roots, stems, and seeds of ARE, TCE, and TFGE (100 g each) were ground into powder in a grinder and extracted with a Soxhlet apparatus with 95% ethanol. The extracts were concentrated via a rotary evaporator at 45°C and dried in a vacuum oven at 40°C [26]. The dried extracts were collected and stored in a closed container in a cool and dry place for future use. A blend of all three extracts at a ratio of 1:1:1 w/w/w (ATTEB) was prepared and used for further studies.

### 2.3. Phytochemical Analysis of Extracts

The individual extracts and ATTEB were subjected to preliminary phytochemical investigations [27–29].

### 2.4. Experimental Animals

Swiss albino mice (23–28 g body weight) were housed in an institutional animal facility at a constant temperature (22 ± 3°C) and humidity (45%–55%). A light/dark cycle of 12 h was maintained throughout the trial. The animals were fasted for 4 h before dosing. The food, but not the water, was restricted for 2 h daily after dosing. During the research, standard pellet feed was provided to the animals. The Institutional Animal Ethics Committee (IAEC) of IAHVB, M.P., India, constituted by the Committee for Control and Supervision of Experiments on Animals (CCSEA), Government of India, New Delhi, approved all the study protocols (approval no. IAEC/2019/01/02) and supervised the complete work for compliance with ethical requirements as per the guidelines [30]. The 14-day acute toxicity assessment and 28-day repeated-dose safety assessment of ATTEB were conducted in full compliance with the Organization of Economic Cooperation and Development (OECD) guidelines 423 and 407 [31, 32].

The extracts were administered orally via gavage at the respective dosages in the studies.

#### 2.4.1. Dose Preparation

A mixture of dried ethanolic extracts of ARE, TCE, and TFGE at a 1:1:1 ratio (ATTEB) was prepared in distilled water. A maximum of 1 mL/100 g body weight was administered to experimental animals [33]. The doses for toxicity studies were prepared shortly prior to administration.

### 2.5. Acute Toxicity Studies

The acute toxicity tests were conducted according to OECD guidelines 423 [31]. The albino mice were separated into three groups comprising three female mice each. Only on the first day of the trial did Group 1 receive vehicle (purified drinking water), whereas Groups 2 and 3 received 300 and 2000 mg/kg BW ATTEB, respectively, orally. An Irwin battery analysis (Functional Observational Battery [FOB]) was carried out to establish the behavioral response of the animals in the open cage, home cage, and hand-held conditions for the initial 24 h and weekly for 14 days. Parameters such as mortality, respiration, sedation, body posture, diarrhea, drowsiness, skin color, fur condition, and loss of consciousness (coma) were monitored. The body weight and food and water consumption of the animals were recorded weekly from the first day of the study to 14 days [34]. The animals were sacrificed at the end of the study, and gross necroscopy was performed. Vital organs were isolated, weighed, and observed for morphology [35].

### 2.6. Repeated-Dose Toxicity Studies (Subacute Toxicity Study)

The OECD Guideline 407 mandates that repeated-dose toxicity studies be conducted [32]. Forty Swiss albino mice (25–30 g, 20 males and 20 females) were sorted into four groups of five males and five females each. Polypropylene cages were used to house the animals for the duration of the experiments. Each day for 28 days, pure water and ATTEB at doses of 250, 500, and 1000 mg/kg of body weight were administered to the mice in Groups 1–4, respectively. On the final day of the study, the animals were sacrificed, and their blood was collected for biochemical analysis (Star 21 Plus, Rapid Diagnostic Pvt. Ltd., India) and hematological studies (Prokan Fully Automatic PE 6800 VET Hematology Analyzer, China) [34]. Organs were removed from each animal in the study and compared with the control group's organs in terms of weight and histological analysis. A Motic DMB1 Digital Upright Microscope (India) was used to examine the slides, which were cut with a Microtome (precision rotator microtome) from Scientech, Japan [36, 37].

### 2.7. Micronucleus Test

The micronucleus test was performed via the standard methodology. In brief, 5 groups were constituted (n = 5), viz. Group 1: untreated vehicle control; Groups 2, 3, and 4: the animals were treated with 250, 500, and 1000 mg ATTEB/kg po, respectively; and Group 5: cyclophosphamide 100 mg/kg/animal i.p. Each animal was sacrificed after 30 h postlast dose [38–40]. Bone marrow sampling, slide preparation, and micronucleated polychromatic erythrocyte scoring were performed as described previously. In brief, in fetal calf serum, the bone marrow was flushed and centrifuged (1000 rpm, 5 min), and the resulting pellet was dispersed again in 0.25 mL of fetal calf serum. Smears of the same size were prepared and fixed with 70% methanol. The air-dried slides were subjected to the May–Grunwald–Giemsa staining protocol. A total of 2000 polychromatic erythrocytes were scored per slide from each group.

### 2.8. Statistical Analysis

To compare the means across rows of replicates, we employed one-way analysis of variance (ANOVA) on the toxicity data and then Dunnett's multiple comparison post hoc test. Statistical analysis was performed using Graph Pad Prism 5.0 (San Diego, CA, USA), and the results are presented as the means ± SDs (*n* = 3/5). Statistical significance was defined as a value of ⁣^∗^*p* < 0.05, ⁣^∗∗^*p* < 0.01, or ⁣^∗∗∗^*p* < 0.001 using the Bonferroni correction.

## 3. Results

The percentages of extracts obtained were 10%, 10%, and 12.5% for ARE roots, TCE stems, and TFGEseeds, respectively. A blend of all three extracts at a ratio of 1:1:1 w/w/w (ATTEB) was prepared and used for further studies.

### 3.1. Phytochemical Screening of Plant Extracts

To identify the phytochemicals present in the obtained extracts and ATTEB, a preliminary phytochemical analysis was performed. The results confirmed the presence of the respective bioactive ingredients in the extracts and ATTEB. The detailed observations are given in Table 1.

### 3.2. The Safety Profile of the ATTEB

#### 3.2.1. Acute Toxicity Studies

**Figure 2:**
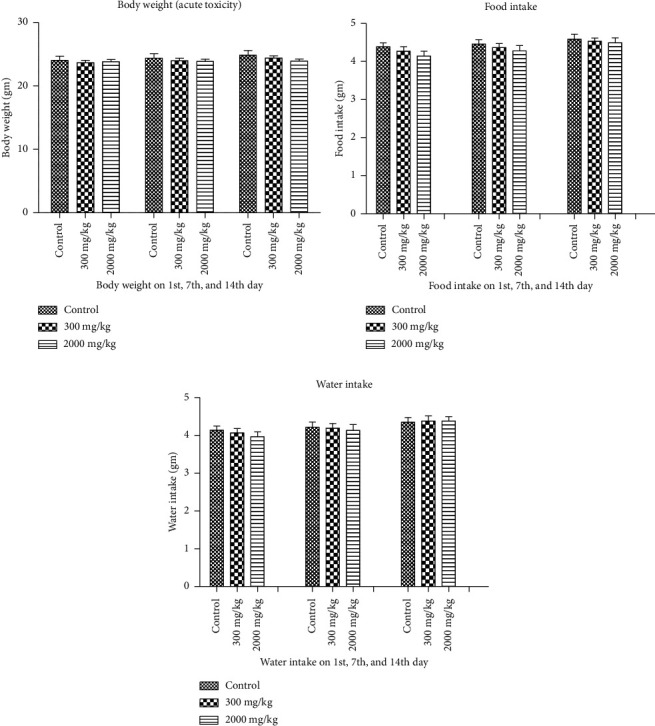
Changes in (a) body weight of control and ATTEB-treated mice at single doses of 300 mg/kg and 2000 mg/kg during the acute toxicity study. The values are expressed as the means ± SDs (*n* = 3). Changes in (b) food intake and (c) water intake of control and ATTEB-treated mice at single doses of 300 mg/kg and 2000 mg/kg during the acute toxicity study. The values are expressed as the means ± SDs (*n* = 3). No significant weight gain was observed after acute treatment with ATTEB for 14 days.

Acute toxicity studies were conducted on the prepared formulation according to OECD recommendations to ensure its safety. The animals were monitored for 14 days after receiving ATTEB. Several measurements were taken. Three groups of three female mice each were given an oral dose of purified water, 300 or 2000 mg/kg BW ATTEB, and observed for 14 days for evidence of toxicity or mortality as part of an acute toxicity test. As a result, we can assume that the LD_50_ of ATTEB is greater than 2000 mg/kg BW. The globally harmonized system (GHS) for the classification and labeling of nonhazardous chemicals with LD50 values above 2000 mg/kg BW was used. Furthermore, all single-dose administrations did not result in a decrease in body weight (Figure 2(a)), and microscopic analysis revealed no evidence of any obvious pathological changes in important internal organs. The following measurements were taken for comparison.

##### 3.2.1.1. Body Weight, Food, and Water Intake

The weight, diet, and water consumption of the animals were tracked. No significant differences in animal body weight were found between the studied groups. There was also no noticeable change in the amount of food or water the animals consumed (Figures 2(a), 2(b), and 2(c)).

##### 3.2.1.2. Cage-Side Observations for 14 days

The mice were either left untreated or given a single dose of ATTEB. After 14 days of surveillance following acute oral therapy with ATTEB, no adverse symptoms or fatalities were observed in the mice (Tables 2 and 3). In addition, no behavioral abnormalities or deaths were observed during the 2-week research period, suggesting that ATTEB is safe. There were no statistically significant differences (*p* > 0.05) in weight gain or loss between the treated and untreated animals. The drug blend-treated animals consumed roughly the same amount of food as the control animals did. The water consumption of the treatment group was comparable with that of the control group. No significant differences in mouse behavior were detected via functional behavior battery measurements between the day of treatment and once-weekly observation or between the control group and experimental groups.

##### 3.2.1.3. Gross Necroscopy of the Animal

After 14 days of observation in the acute toxicity study, the animals were euthanized, and vital organs such as the liver, heart, lung, brain, and kidney were collected. The organs were observed for visual abnormalities in the gross morphology and structure of the organs. Organs were processed by weight and compared with those in the control group. There was no significant difference in the morphology or weight of the organs compared with those of the control group (Table 4).

#### 3.2.2. Repeated-Dose Toxicity Studies

A repeated-dose toxicity study was conducted following OECD recommendations (Guidelines 407) to determine the long-term and multiple-dose safety of the drug. In a 28-day oral toxicity study, no animals died after receiving 250, 500, or 1000 mg/kg BW ATTEB. The levels of toxicity were measured across several dimensions. Throughout the testing and rehabilitation phases, no symptoms of toxicity manifested themselves. No significant clinical signs were detected at any of the three dosages tested in the repeated-dose, 28-day oral toxicity study. The treatment and control groups presented no discernible differences in behavior.

##### 3.2.2.1. Histopathology of Organs

**Figure 3:**
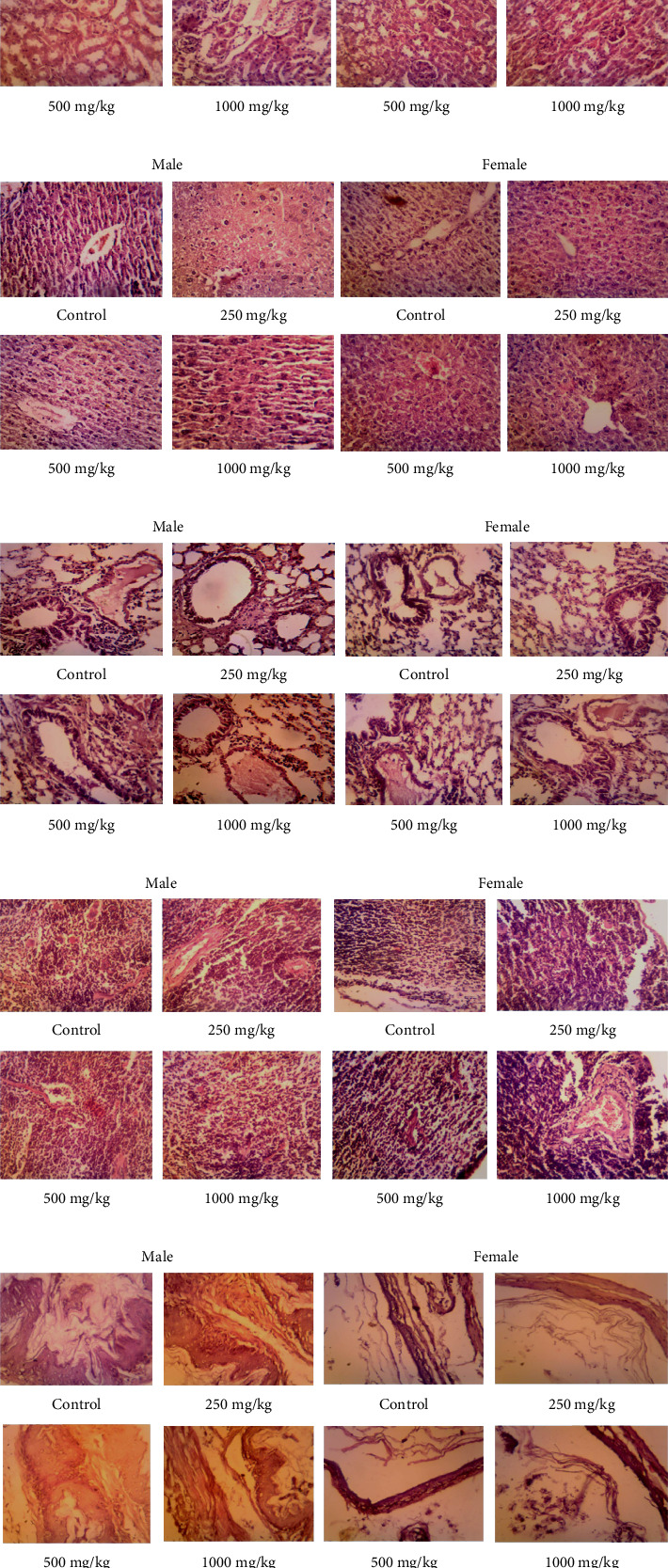
Histopathology of various organs ((a) brain, (b) heart, (c) kidney, (d) liver, (e) lung, (f) spleen, (g) stomach, (h) ovary/testes, and (i) adrenal gland): control group (a), groups treated with 250 mg/kg (b), 500 mg/kg (c), and 1000 mg/kg ATTEB (d). No histopathological alterations were observed in the livers of 1000 mg/kg (repeated-dose) ATTEB-treated mice compared with those of the control group (magnification ×45).

After the repeated dosage toxicity trial, the major organs were subjected to a histological investigation. Figures 3(a), 3(b), 3(c), 3(d), 3(e), 3(f), 3(g), 3(h), and 3(i) show typical photomicrographs of organs from both untreated and ATTEB-treated animals. To observe whether any microscopic changes occurred due to the treatment, the histopathology of vital and other organs defined by regulatory bodies was checked for any microscopic changes. H&E staining of the liver revealed typical hepatic architecture, hepatocytes, and perilobular veins [35]. The glomerulus and tubules in the kidneys of the treated groups seemed normal under the microscope. Cross-sections of hearts from the treated groups revealed healthy myocardial architecture. Sections of the lungs seemed normal, with unremarkable bronchioles and alveoli. The spleen portions appeared normal and included lymphoid follicles and sinuses. The sperm and seminiferous tubules in the testicular portions of the treated groups appeared normal. In conclusion, the subacute study period was not associated with the development of any major pathological organ abnormalities, as evidenced by the results of the histological assay [41].

### 3.3. Body Weight and Relative Organ Weight

**Figure 4:**
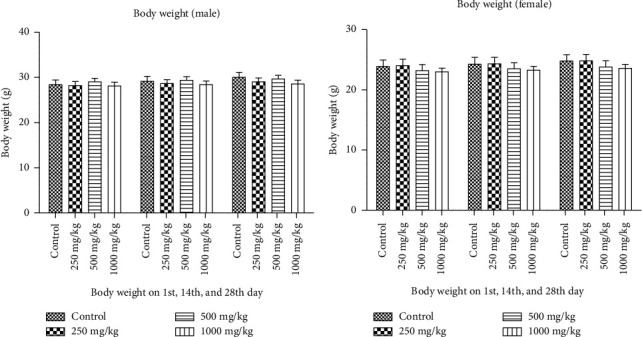
Effects of ATTEB (250, 500, and 1000 mg/kg) on the body weights of (a) male animals and (b) female animals on the 1^st^, 14^th^, and 28^th^ days of the repeated-dose toxicity study. The values are expressed as the means ± SDs and were analyzed via one-way ANOVA followed by Dunnett's test (*n* = 5 animals/group). No significant weight change was observed in the body weights of male and female mice after repeated-dose treatment with ATTEB for 28 days.

Compared with their respective control groups, male and female mice given the medication mixture daily for 28 days presented no significant differences in body weight. In addition, there was no statistically significant difference in organ weight between the experimental and control groups (*p* < 0.001) (Figure 4).

### 3.4. Biochemical Analysis

#### 3.4.1. Effects on Serum Marker Enzymes During the 28-Day Treatment

The serum levels of ALT, AST, and ALP in both sexes did not significantly differ from those in their respective control groups across any dose range (Table 5).

#### 3.4.2. Effects on Total Serum Protein, Urea, and Creatinine Levels

The total protein levels in the serum of male and female mice were not significantly different from those in the control after 28 days of treatment with any dose of the drug blend (Table 5). In addition, there were no notable differences in the concentrations of blood urea nitrogen and creatinine between the sexes (Table 5).

#### 3.4.3. Effects on the Lipid Profile

The serum triglyceride and total cholesterol values after medication blend therapy are shown in Table 5 for both male and female mice.

#### 3.4.4. Hematological and Biochemical Parameters

The effects of ATTEB on the general biochemical parameters of the animals after 28 days of daily treatment are shown in Table 5. When the hematological parameters of the treated animals were compared with those of the control animals, no significant differences were found (Table 6). The treated mice showed no abnormalities in their biochemical markers. The serum electrolyte levels were not significantly different from those in the control group. Tests for acute and repeated toxicity in mice revealed no obvious toxicity from ATTEB.

### 3.5. Genotoxicity

**Figure 5:**
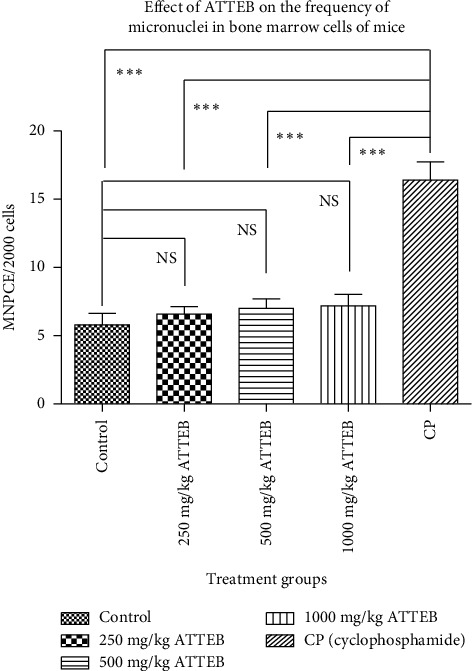
Effects of ATTEB (250, 500, and 1000) on mouse bone marrow cells. The values are expressed as the means ± SDs and were analyzed via one-way ANOVA followed by Dunnett's test (*n* = 5 animals/group). Statistical significance was defined as a value of ⁣^∗^*p* < 0.05, ⁣^∗∗^*p* < 0.01, or ⁣^∗∗∗^*p* < 0.001. A significant difference in the presence of micronuclei in bone marrow cells was detected between the control and ATTEB-treated groups and the cyclophosphamide-treated group.

After treatment with ATTEB, MNPCE were counted to assess micronucleus development due to cleavage of the chromosomes. A significantly greater number of micronuclei were detected in the cyclophosphamide-treated group than those in the control and ATTEB-treated groups (Figure 5). The results indicated that the drug does not have any genotoxicity potential at the level of 1000 mg/kg ATTEB.

## 4. Discussion

Herbal drugs and traditional medicines have been used in developing countries owing to their safety and effectiveness. In the rural population of a developing country, these drugs are often self-prepared and self-medicated. It is widely used orally for the prevention and treatment of a variety of diseases as well as for the promotion of wellbeing. The dose is rarely monitored, and thus, many times, it is taken for an extensive period, owing to the understanding that these are free from any side effects or adverse effects. Therefore, well-designed scientific studies are highly desirable for such herbal and traditional medicines [42, 43].

The currently studied ATTEB is a popular herbal remedy that is used for a variety of diseases [44]. There are several proprietary medicines available on the market that contain these herbs along with other herbal combinations. This approach is widely used as a self-prepared and self-medicated combination. A systematic study of this popular herbal medicine is warranted, considering its safety concerns to protect consumers from potential adverse effects. A preliminary study to assess its pharmacological activity is to study its safety profile. Therefore, to determine the NOAEL and any target organ toxicity, the current study of the ATTEB was carried out as per the standard guidelines for oral acute toxicity studies, that is, OECD 423, and the repeated-dose 28-day oral toxicity study, that is, OECD 407.

The extracts obtained from the selected plant material and ATTEB were subjected to preliminary phytochemical analysis and found to contain biochemicals such as carbohydrates, alkaloids, proteins and amino acids, tannins and phenolics, saponins, flavonoids, glycosides, and mucilage.

Furthermore, in this study, a single dose of ATTEB at doses of 300 and 1000 mg/kg BW in mice was administered and found to be safe. The administered dose did not cause any mortality, changes in water intake, consumption of food, or body weight. It may affect an animal's overall wellbeing, routine activities, energy production, and behavior, which may be reflected through the assessment of Irwin battery parameters. There was no significant change in the parameters of the Irwin battery. A toxic dose may adversely affect inner organs, principally vital organs, and may lead to alterations in their weight and appearance. Thus, gross necropsy was performed, and individual organs of the animal were harvested. Compared with those of the control group, no significant changes in the size or shape of the organs were observed. Compared with that of the control organs, the weight of individual organs was found to be normal. The animals were administered the highest dose of 2000 mg/kg BW for the acute toxicity study, and all the animals survived the dose, confirming that the LD_50_ of ATTEB is greater than that of 2000 mg/kg BW. Moreover, no treatment-related clinical emergencies, signs of disease, or abnormalities were observed, which indicates that ATTEB is safe at the given dose in animals [45].

To acquire a wider spectrum of information on the health effects of ATTEB on mice, repeated-dose toxicity study was performed. On the basis of these findings, the doses for repeated-dose toxicity were selected as low, intermediate, and high doses, that is, 250, 500, and 1000 mg/kg BW. The span of 28 days is a considerable amount of time spent by the mice to affect their overall wellbeing. The administration of the drug for 28 days affects animal physiology and may modulate homeostasis. In the 28-day study, an increase in age and food intake with time may have contributed to an increase in body weight, which was also synchronous with the findings of the control group. Therefore, it can be inferred that ATTEB does not interfere with the metabolism of the animal [45, 46]. The ATTEB did not alter the weight of the vital or visceral organs at a subacute dose.

Orally administered toxins are absorbed through the intestine and enter the blood. Blood components are among the most sensitive targets for such chemicals and are easily modulated. Moreover, blood is the most important part of the body because it maintains homeostasis in terms of temperature, energy, hydration, oxygenation of tissues, removal of waste material, and immune response. Therefore, the assessment of hematological parameters has become vital for assessing toxicity. ATTEB did not affect hematological parameters, indicating that there was no significant physiological alteration in in vivo homeostasis. The white blood cell count, hemoglobin, red blood cell count, mean corpuscular volume, packed cell volume, mean corpuscular hemoglobin, and mean corpuscular hemoglobin concentration were recorded to be within the normal limits in the control and all the treatment groups, indicating that there was no significant effect of treatment on hematological parameters.

Furthermore, the signs and symptoms of any disease or disorder are produced due to the modulation of biochemical parameters in the body. These parameters are biomarkers of the function of vital organs and organ systems. All the principal vital biochemical markers were checked for any possible modulation, and the results indicated that ATTEB did not significantly modulate any biochemical markers. Toxic chemicals are metabolized by the liver and excreted as waste material from the kidney. Thus, they intensely interact with the liver and kidney, affecting their cells and functions. Thus, to analyze the normal functioning of these organs, their respective biomarkers were analyzed. The biochemical analysis of creatinine, blood urea nitrogen, and blood electrolytes revealed that there was no adverse effect of ATTEB on the pre, post, or inside kidney tubules. Normal levels of ALT, AST, and ALP indicate the normal status of liver hepatocytes in both sexes [47]. There was no significant change in the levels of serum triglycerides or total cholesterol in male or female mice. Thus, these findings reveal the nontoxic nature of ATTEB in the animal body.

Histopathological analysis of vital and visceral organs revealed no vital organ toxicity or reproductive toxicity. The biochemical parameters of organ biomarkers support the histopathological findings [37].

In the present study, the genotoxicity potential of ATTEB was also evaluated by performing in vivo micronucleus assay in a mouse model. The drug cyclophosphamide was used as a standard toxicant to induce micronuclei in mouse bone marrow cells. As per the current regulations, all drug candidates should be tested for genotoxicity before efficacy studies. The herbal drug is free from genotoxicity in vivo and can be considered safe for further development as a drug (Figure 5).

The results of acute and repeated-dose toxicity indicated that ATTEB did not have any noticeable toxicity in mice.

## 5. Conclusion

The oral acute toxicity (OECD 423 guidelines) studies at doses up to 2000 mg/kg BW have shown no mortality and no observable signs of toxicity in mice. Food and water intake were not affected; there were no abnormalities in behavior (in a cage, out of the cage, or while handling), and gross necroscopic analysis also revealed no adverse effects on the mice. Furthermore, as ATTEB has an LD_50_ greater than 2000 mg/kg BW, it can be included as a category 4 chemical, as described by the OECD. Moreover, various behavioral, hematological, and biochemical parameters post-28-day repeated-dose toxicity (OECD 407) studies with 250, 500, and 1000 mg/kg BW ATTEB were found to be within a safe limit compared with those of the control group. Histopathological studies of various vital and other visceral organs revealed no observable microscopic abnormalities after 28 days of repeated-dose studies. These findings indicate that ATTEB is safe for further clinical studies and the development of formulations for the prevention and treatment of various ailments.

## Figures and Tables

**Table 1:** Phytochemical characterization of plant materials.

S. no.	Phytoconstituents	*A. racemosus*	*T. cordifolia*	*T. foenum-graecum*	ATTEB
1	Carbohydrates	+	+	+	+
2	Alkaloids	−	+	+	+
3	Proteins and amino acids	−	+	−	+
4	Tannins and phenolics	−	+	+	+
5	Saponins	+	−	+	+
6	Flavonoids	−	+	+	+
7	Triterpenoids	−	−	−	−
8	Steroids	−	−	−	−
9	Glycosides	+	+	+	+
10	Fixed oils	−	−	−	−
11	Gums	−	−	−	−
12	Mucilage	+	−	−	+

**Table 2:** Functional behavioral battery parameters on the day of dosing of control and ATTEB-treated mice at single doses of 300 and 2000 mg/kg BW.

Categories	Control								ATTEB treated (300 mg/kg BW)								ATTEB treated (2000 mg/kg BW)							
	0 m	30 m	1 h	2 h	4 h	8 h	12 h	24 h	0 m	30 m	1 h	2 h	4 h	8 h	12 h	24 h	0 m	30 m	1 h	2 h	4 h	8 h	12 h	24 h
Home cage																								
Spontaneous activity level	No	No	No	No	No	No	No	No	No	No	No	No	No	No	No	No	No	No	No	No	No	No	No	No
Posture	No	No	No	No	No	No	No	No	No	No	No	No	No	No	No	No	No	No	No	No	No	No	No	No
Respiration	No	No	No	No	No	No	No	No	No	No	No	No	No	No	No	No	No	No	No	No	No	No	No	No
Convulsions	Ab	Ab	Ab	Ab	Ab	Ab	Ab	Ab	Ab	Ab	Ab	Ab	Ab	Ab	Ab	Ab	Ab	Ab	Ab	Ab	Ab	Ab	Ab	Ab
Tremors	Ab	Ab	Ab	Ab	Ab	Ab	Ab	Ab	Ab	Ab	Ab	Ab	Ab	Ab	Ab	Ab	Ab	Ab	Ab	Ab	Ab	Ab	Ab	Ab
Fasciculations	Ab	Ab	Ab	Ab	Ab	Ab	Ab	Ab	Ab	Ab	Ab	Ab	Ab	Ab	Ab	Ab	Ab	Ab	Ab	Ab	Ab	Ab	Ab	Ab
Tonus	Ng	Ng	Ng	Ng	Ng	Ng	Ng	Ng	Ng	Ng	Ng	Ng	Ng	Ng	Ng	Ng	Ng	Ng	Ng	Ng	Ng	Ng	Ng	Ng
Clonus	Ng	Ng	Ng	Ng	Ng	Ng	Ng	Ng	Ng	Ng	Ng	Ng	Ng	Ng	Ng	Ng	Ng	Ng	Ng	Ng	Ng	Ng	Ng	Ng
Vocalization	Pt	Pt	Pt	Pt	Pt	Pt	Pt	Pt	Pt	Pt	Pt	Pt	Pt	Pt	Pt	Pt	Pt	Pt	Pt	Pt	Pt	Pt	Pt	Pt
Straub tail	Ab	Ab	Ab	Ab	Ab	Ab	Ab	Ab	Ab	Ab	Ab	Ab	Ab	Ab	Ab	Ab	Ab	Ab	Ab	Ab	Ab	Ab	Ab	Ab
Writhing	Ab	Ab	Ab	Ab	Ab	Ab	Ab	Ab	Ab	Ab	Ab	Ab	Ab	Ab	Ab	Ab	Ab	Ab	Ab	Ab	Ab	Ab	Ab	Ab
Retropulsion	Ab	Ab	Ab	Ab	Ab	Ab	Ab	Ab	Ab	Ab	Ab	Ab	Ab	Ab	Ab	Ab	Ab	Ab	Ab	Ab	Ab	Ab	Ab	Ab
Diarrhea	Ab	Ab	Ab	Ab	Ab	Ab	Ab	Ab	Ab	Ab	Ab	Ab	Ab	Ab	Ab	Ab	Ab	Ab	Ab	Ab	Ab	Ab	Ab	Ab

Hand held																								
Excitation	Ab	Ab	Ab	Ab	Ab	Ab	Ab	Ab	Ab	Ab	Ab	Ab	Ab	Ab	Ab	Ab	Ab	Ab	Ab	Ab	Ab	Ab	Ab	Ab
Salivation	Ab	Ab	Ab	Ab	Ab	Ab	Ab	Ab	Ab	Ab	Ab	Ab	Ab	Ab	Ab	Ab	Ab	Ab	Ab	Ab	Ab	Ab	Ab	Ab
Lacrimation	Ab	Ab	Ab	Ab	Ab	Ab	Ab	Ab	Ab	Ab	Ab	Ab	Ab	Ab	Ab	Ab	Ab	Ab	Ab	Ab	Ab	Ab	Ab	Ab
Piloerection	Ab	Ab	Ab	Ab	Ab	Ab	Ab	Ab	Ab	Ab	Ab	Ab	Ab	Ab	Ab	Ab	Ab	Ab	Ab	Ab	Ab	Ab	Ab	Ab
Fur appearance	No	No	No	No	No	No	No	No	No	No	No	No	No	No	No	No	No	No	No	No	No	No	No	No
Eye	Ab	Ab	Ab	Ab	Ab	Ab	Ab	Ab	Ab	Ab	Ab	Ab	Ab	Ab	Ab	Ab	Ab	Ab	Ab	Ab	Ab	Ab	Ab	Ab

Open cage																								
Supported rears	Ab	Ab	Ab	Ab	Ab	Ab	Ab	Ab	Ab	Ab	Ab	Ab	Ab	Ab	Ab	Ab	Ab	Ab	Ab	Ab	Ab	Ab	Ab	Ab
Unsupported rears	Ab	Ab	Ab	Ab	Ab	Ab	Ab	Ab	Ab	Ab	Ab	Ab	Ab	Ab	Ab	Ab	Ab	Ab	Ab	Ab	Ab	Ab	Ab	Ab
Spontaneous activity level	No	No	No	No	No	No	No	No	No	No	No	No	No	No	No	No	No	No	No	No	No	No	No	No
Gait	No	No	No	No	No	No	No	No	No	No	No	No	No	No	No	No	No	No	No	No	No	No	No	No
Posture	No	No	No	No	No	No	No	No	No	No	No	No	No	No	No	No	No	No	No	No	No	No	No	No
Arousal	No	No	No	No	No	No	No	No	No	No	No	No	No	No	No	No	No	No	No	No	No	No	No	No
Convulsions	Ab	Ab	Ab	Ab	Ab	Ab	Ab	Ab	Ab	Ab	Ab	Ab	Ab	Ab	Ab	Ab	Ab	Ab	Ab	Ab	Ab	Ab	Ab	Ab
Straub tail	Ab	Ab	Ab	Ab	Ab	Ab	Ab	Ab	Ab	Ab	Ab	Ab	Ab	Ab	Ab	Ab	Ab	Ab	Ab	Ab	Ab	Ab	Ab	Ab
Writhing	Ab	Ab	Ab	Ab	Ab	Ab	Ab	Ab	Ab	Ab	Ab	Ab	Ab	Ab	Ab	Ab	Ab	Ab	Ab	Ab	Ab	Ab	Ab	Ab
Retropulsion	Ab	Ab	Ab	Ab	Ab	Ab	Ab	Ab	Ab	Ab	Ab	Ab	Ab	Ab	Ab	Ab	Ab	Ab	Ab	Ab	Ab	Ab	Ab	Ab
Stereotypy	Ab	Ab	Ab	Ab	Ab	Ab	Ab	Ab	Ab	Ab	Ab	Ab	Ab	Ab	Ab	Ab	Ab	Ab	Ab	Ab	Ab	Ab	Ab	Ab
Diarrhea	Ab	Ab	Ab	Ab	Ab	Ab	Ab	Ab	Ab	Ab	Ab	Ab	Ab	Ab	Ab	Ab	Ab	Ab	Ab	Ab	Ab	Ab	Ab	Ab
Auditory response	Pt	Pt	Pt	Pt	Pt	Pt	Pt	Pt	Pt	Pt	Pt	Pt	Pt	Pt	Pt	Pt	Pt	Pt	Pt	Pt	Pt	Pt	Pt	Pt
Somatosensory response	Pt	Pt	Pt	Pt	Pt	Pt	Pt	Pt	Pt	Pt	Pt	Pt	Pt	Pt	Pt	Pt	Pt	Pt	Pt	Pt	Pt	Pt	Pt	Pt
Visual approach	Pt	Pt	Pt	Pt	Pt	Pt	Pt	Pt	Pt	Pt	Pt	Pt	Pt	Pt	Pt	Pt	Pt	Pt	Pt	Pt	Pt	Pt	Pt	Pt
Olfactory response	Pt	Pt	Pt	Pt	Pt	Pt	Pt	Pt	Pt	Pt	Pt	Pt	Pt	Pt	Pt	Pt	Pt	Pt	Pt	Pt	Pt	Pt	Pt	Pt
Pinna reflex	Pt	Pt	Pt	Pt	Pt	Pt	Pt	Pt	Pt	Pt	Pt	Pt	Pt	Pt	Pt	Pt	Pt	Pt	Pt	Pt	Pt	Pt	Pt	Pt
Extensor reflex	Pt	Pt	Pt	Pt	Pt	Pt	Pt	Pt	Pt	Pt	Pt	Pt	Pt	Pt	Pt	Pt	Pt	Pt	Pt	Pt	Pt	Pt	Pt	Pt
Palpebral reflex	Pt	Pt	Pt	Pt	Pt	Pt	Pt	Pt	Pt	Pt	Pt	Pt	Pt	Pt	Pt	Pt	Pt	Pt	Pt	Pt	Pt	Pt	Pt	Pt
Visual placing	Pt	Pt	Pt	Pt	Pt	Pt	Pt	Pt	Pt	Pt	Pt	Pt	Pt	Pt	Pt	Pt	Pt	Pt	Pt	Pt	Pt	Pt	Pt	Pt
Righting	Ab	Ab	Ab	Ab	Ab	Ab	Ab	Ab	Ab	Ab	Ab	Ab	Ab	Ab	Ab	Ab	Ab	Ab	Ab	Ab	Ab	Ab	Ab	Ab
Pupil reaction	No	No	No	No	No	No	No	No	No	No	No	No	No	No	No	No	No	No	No	No	No	No	No	No
Tail pinch response	Pt	Pt	Pt	Pt	Pt	Pt	Pt	Pt	Pt	Pt	Pt	Pt	Pt	Pt	Pt	Pt	Pt	Pt	Pt	Pt	Pt	Pt	Pt	Pt
Urination spots	Ab	Ab	Ab	Ab	Ab	Ab	Ab	Ab	Ab	Ab	Ab	Ab	Ab	Ab	Ab	Ab	Ab	Ab	Ab	Ab	Ab	Ab	Ab	Ab
Mortality	Ab	Ab	Ab	Ab	Ab	Ab	Ab	Ab	Ab	Ab	Ab	Ab	Ab	Ab	Ab	Ab	Ab	Ab	Ab	Ab	Ab	Ab	Ab	Ab

Abbreviations: Ab, absent; Ng, negative; No, normal; Pt, present.

**Table 3:** Functional behavioral battery parameters observed once a week in control and ATTEB-treated mice at single doses of 300 and 2000 mg/kg BW.

Categories	Control			ATTEB treated (300 mg/kg BW)			ATTEB treated (2000 mg/kg BW)		
	Day 1	Day 7	Day 14	Day 1	Day 7	Day 14	Day 1	Day 7	Day 14
Home cage									
Spontaneous activity level	No	No	No	No	No	No	No	No	No
Posture	No	No	No	No	No	No	No	No	No
Respiration	No	No	No	No	No	No	No	No	No
Convulsions	Ab	Ab	Ab	Ab	Ab	Ab	Ab	Ab	Ab
Tremors	Ab	Ab	Ab	Ab	Ab	Ab	Ab	Ab	Ab
Fasciculations	Ab	Ab	Ab	Ab	Ab	Ab	Ab	Ab	Ab
Tonus	Ng	Ng	Ng	Ng	Ng	Ng	Ng	Ng	Ng
Clonus	Ng	Ng	Ng	Ng	Ng	Ng	Ng	Ng	Ng
Vocalization	Pt	Pt	Pt	Pt	Pt	Pt	Pt	Pt	Pt
Straub tail	Ab	Ab	Ab	Ab	Ab	Ab	Ab	Ab	Ab
Writhing	Ab	Ab	Ab	Ab	Ab	Ab	Ab	Ab	Ab
Retropulsion	Ab	Ab	Ab	Ab	Ab	Ab	Ab	Ab	Ab
Diarrhea	Ab	Ab	Ab	Ab	Ab	Ab	Ab	Ab	Ab

Hand held									
Excitation	Ab	Ab	Ab	Ab	Ab	Ab	Ab	Ab	Ab
Salivation	Ab	Ab	Ab	Ab	Ab	Ab	Ab	Ab	Ab
Lacrimation	Ab	Ab	Ab	Ab	Ab	Ab	Ab	Ab	Ab
Piloerection	Ab	Ab	Ab	Ab	Ab	Ab	Ab	Ab	Ab
Fur appearance	No	No	No	No	No	No	No	No	No
Eye	Ab	Ab	Ab	Ab	Ab	Ab	Ab	Ab	Ab

Open cage									
Supported rears	Ab	Ab	Ab	Ab	Ab	Ab	Ab	Ab	Ab
Unsupported rears	Ab	Ab	Ab	Ab	Ab	Ab	Ab	Ab	Ab
Spontaneous activity level	No	No	No	No	No	No	No	No	No
Gait	No	No	No	No	No	No	No	No	No
Posture	No	No	No	No	No	No	No	No	No
Arousal	No	No	No	No	No	No	No	No	No
Convulsions	Ab	Ab	Ab	Ab	Ab	Ab	Ab	Ab	Ab
Straub tail	Ab	Ab	Ab	Ab	Ab	Ab	Ab	Ab	Ab
Writhing	Ab	Ab	Ab	Ab	Ab	Ab	Ab	Ab	Ab
Retropulsion	Ab	Ab	Ab	Ab	Ab	Ab	Ab	Ab	Ab
Stereotypy	Ab	Ab	Ab	Ab	Ab	Ab	Ab	Ab	Ab
Diarrhea	Ab	Ab	Ab	Ab	Ab	Ab	Ab	Ab	Ab
Auditory response	Pt	Pt	Pt	Pt	Pt	Pt	Pt	Pt	Pt
Somatosensory response	Pt	Pt	Pt	Pt	Pt	Pt	Pt	Pt	Pt
Visual approach	Pt	Pt	Pt	Pt	Pt	Pt	Pt	Pt	Pt
Olfactory response	Pt	Pt	Pt	Pt	Pt	Pt	Pt	Pt	Pt
Pinna reflex	Pt	Pt	Pt	Pt	Pt	Pt	Pt	Pt	Pt
Extensor reflex	Pt	Pt	Pt	Pt	Pt	Pt	Pt	Pt	Pt
Palpebral reflex	Pt	Pt	Pt	Pt	Pt	Pt	Pt	Pt	Pt
Visual placing	Pt	Pt	Pt	Pt	Pt	Pt	Pt	Pt	Pt
Righting	Ab	Ab	Ab	Ab	Ab	Ab	Ab	Ab	Ab
Pupil reaction	No	No	No	No	No	No	No	No	No
Tail pinch response	Pt	Pt	Pt	Pt	Pt	Pt	Pt	Pt	Pt
Urination spots	Ab	Ab	Ab	Ab	Ab	Ab	Ab	Ab	Ab
Mortality	Ab	Ab	Ab	Ab	Ab	Ab	Ab	Ab	Ab

Abbreviations: Ab, absent; Ng, negative; No, normal; Pt, present.

**Table 4:** Absolute organ weights (in g) of control and ATTEB-treated mice at single doses of 300 and 2000 mg/kg BW.

	Group I	Group II	Group III
Body weight	24.85 ± 0.74	24.41 ± 0.33	23.94 ± 0.30
Liver	1.344 ± 0.034	1.310 ± 0.013	1.322 ± 0.004
Heart	0.1644 ± 0.004	0.162 ± 0.002	0.159 ± 0.001
Lung	0.188 ± 0.003	0.186 ± 0.004	0.1843 ± 0.001
Brain	0.349 ± 0.009	0.347 ± 0.010	0.339 ± 0.003
Kidney	0.378 ± 0.007	0.377 ± 0.003	0.369 ± 0.004

*Note:* Value expressed as the mean ± SD, in g. There is no significant (*p* < 0.05) difference between body weight and relative organ weight of control and single-dose ATTEB-treated group for 14 days.

**Table 5:** Biochemical parameters of the mice after administration of 250, 500, or 1000 mg/kg ATTEB for 28 days.

Parameters	Male				Female			
	Group I	Group II	Group III	Group IV	Group I	Group II	Group III	Group IV
BUN (mg/dL)	25.15 ± 1.11	26.388 ± 1.38	24.198 ± 0.90	24.798 ± 0.83	21.612 ± 2.39	23.374 ± 2.83	22.388 ± 1.28	22.916 ± 0.76
Creatinine (mg/dL)	0.428 ± 0.05	0.472 ± 0.06	0.522 ± 0.07	0.514 ± 0.05	0.448 ± 0.05	0.436 ± 0.02	0.458 ± 0.02	0.486 ± 0.03
Glucose (mg/dL)	113.534 ± 5.06	115.892 ± 5.29	109.768 ± 3.97	105.466 ± 1.53	114.642 ± 5.22	112.444 ± 5.15	108.942 ± 2.58	104.328 ± 4.77
Total protein (g/dL)	5.832 ± 0.73	6.18 ± 0.56	5.512 ± 0.47	4.986 ± 0.34	5.562 ± 0.31	5.982 ± 0.39	5.292 ± 0.28	4.99 ± 0.204
ALT (SGPT)(U/L)	42.634 ± 2.56	41.574 ± 1.77	41.382 ± 2.29	42.038 ± 2.19	39.61 ± 2.74	38.13 ± 1.86	37.594 ± 0.63	37.814 ± 0.63
AST (SGOT)(U/L)	121.132 ± 2.16	118.58 ± 2.41	120.794 ± 2.14	120.112 ± 1.45	116.296 ± 2.30	114.228 ± 0.95	119.586 ± 1.20	120.046 ± 2.85
ALP (U/L)	252.356 ± 12.06	263.732 ± 11.92	254.182 ± 13.50	258.778 ± 15.16	276.156 ± 8.91	280.31 ± 6.15	283.868 ± 10.13	286.948 ± 8.62
Total bilirubin (mg/dL)	0.358 ± 0.03	0.29 ± 0.04	0.322 ± 0.05	0.39 ± 0.04	0.386 ± 0.018	0.368 ± 0.032	0.358 ± 0.016	0.406 ± 0.02
Total cholesterol (mg/dL)	97.414 ± 4.52	96.34 ± 2.85	94.37 ± 3.55	92.27 ± 3.64	98.44 ± 3.96	97.478 ± 4.13	94.218 ± 2.56	93.124 ± 2.98
Triglyceride (mg/dL)	58.566 ± 2.61	60.368 ± 3.07	57.38 ± 3.53	55.912 ± 3.27	60.608 ± 2.54	62.568 ± 2.88	61.938 ± 2.24	59.55 ± 2.01
Sodium (mEq/L)	138.238 ± 6.72	131.738 ± 5.17	135.562 ± 4.29	129.542 ± 3.48	130.824 ± 3.43	129.59 ± 1.27	128.754 ± 2.82	127.48 ± 3.19
Potassium (mEq/L)	3.966 ± 0.23	4.13 ± 0.19	4.056 ± 0.15	3.824 ± 0.09	4.072 ± 0.18	4.212 ± 0.1	4.17 ± 0.09	3.984 ± 0.09
Chloride (mEq/L)	103.704 ± 5.16	102.63 ± 3.17	101.46 ± 2.85	98.596 ± 2.69	101.258 ± 1.84	100.238 ± 2.18	98.768 ± 1.14	98.412 ± 1.80

*Note:* Value expressed as the mean ± SD (*n* = 5). No significant (*p* < 0.05) change in biochemical parameters of mice was observed in male and female mice after repeated-dose treatment with ATTEB for 28 days.

**Table 6:** Hematological parameters of the mice after administration of 250, 500, or 1000 mg/kg ATTEB for 28 days.

Parameters	Male				Female			
	Group I	Group II	Group III	Group IV	Group I	Group II	Group III	Group IV
Hb (g/dL)	14.152 ± 0.196	14.356 ± 0.21	14.166 ± 0.15	13.982 ± 0.16	13.326 ± 0.34	13.572 ± 0.31	13.672 ± 0.29	13.488 ± 0.17
RBC (∗10^6^/*μ*L)	10.954 ± 0.21	10.366 ± 0.056	10.296 ± 0.093	10.21 ± 0.048	9.474 ± 0.49	9.81 ± 0.45	9.624 ± 0.31	9.582 ± 0.26
WBC (∗10^3^/*μ*L)	8.85 ± 0.4	8.746 ± 0.33	8.93 ± 0.118	9.014 ± 0.214	7.44 ± 1.18	7.634 ± 0.96	7.816 ± 1.18	8.326 ± 0.92
Platelets (∗10^3^/*μ*L)	489.8 ± 20.51	516.4 ± 19.82	472.6 ± 21.38	508 ± 17.56	458.4 ± 15.49	478.8 ± 12.39	446.6 ± 12.93	467 ± 15.62
Basophils (%)	0.26 ± 0.03	0.246 ± 0.02	0.266 ± 0.02	0.252 ± 0.02	0.224 ± 0.06	0.216 ± 0.06	0.218 ± 0.07	0.214 ± 0.05
Lymphocytes (%)	74.932 ± 4.305	74.414 ± 4.1	78.67 ± 4.87	81.582 ± 3.89	71.894 ± 2.95	73.81 ± 2.64	76.566 ± 2.75	79.462 ± 2.14
Monocytes (%)	1.128 ± 0.12	1.178 ± 0.06	1.19 ± 0.05	1.15 ± 0.1	1.03 ± 0.07	1.06 ± 0.06	1.056 ± 0.05	1.04 ± 0.04
MCV (fL)	46.292 ± 2.35	48.192 ± 2.66	49.796 ± 2.59	49.964 ± 2.74	42.222 ± 1.63	44.216 ± 1.13	44.974 ± 2.16	45.184 ± 2.12
MCHC (%)	35.392 ± 1.11	36.156 ± 1.42	36.692 ± 1.08	35.728 ± 1.76	33.258 ± 0.69	33.772 ± 0.31	33.828 ± 0.26	32.788 ± 0.33
MCH (pg)	14.478 ± 0.386	14.612 ± 0.25	14.868 ± 0.18	14.63 ± 0.15	14.538 ± 0.56	14.826 ± 0.51	15.036 ± 0.42	14.868 ± 0.21
Eosinophils (%)	2.352 ± 0.087	2.316 ± 0.04	2.376 ± 0.05	2.342 ± 0.04	2.386 ± 0.16	2.332 ± 0.11	2.394 ± 0.13	2.404 ± 0.10
Neutrophils (%)	23.61 ± 1.55	21.76 ± 1.22	22.51 ± 1.19	23.386 ± 1.09	21.63 ± 2.06	20.97 ± 0.98	21.74 ± 0.99	22.288 ± 1.00

*Note:* Value expressed as the mean ± SD (*n* = 5). No significant (*p* < 0.05) change in hematological parameters of mice was observed in male and female mice after repeated-dose treatment with ATTEB for 28 days.

## Data Availability

The data used to support the findings of this study are available from the corresponding author upon reasonable request.
